# Pre-operative urinary cathepsin D is associated with survival in patients with renal cell carcinoma

**DOI:** 10.1038/sj.bjc.6605250

**Published:** 2009-09-29

**Authors:** N S Vasudev, S Sim, D A Cairns, R E Ferguson, R A Craven, A Stanley, J Cartledge, D Thompson, P J Selby, R E Banks

**Affiliations:** 1 Cancer Research UK Clinical Centre, Leeds Institute of Molecular Medicine, St James's University Hospital, Leeds, UK; 2 Pyrah Department of Urology, St James's University Hospital, Leeds, UK; 3 Department of Clinical Biochemistry and Immunology, St James's University Hospital, Leeds, UK

**Keywords:** renal cell carcinoma, cathepsin D, urine, prognosis, biomarker, proteomics

## Abstract

**Background:**

No circulating markers are routinely used for renal cancer. The objective of this pilot study was to investigate whether conditioned media (CM) from renal cancer cell lines contains potential biomarkers that, when measured in clinical fluids, have diagnostic or prognostic utility.

**Methods:**

Comparative 2D PAGE profiling of CM from renal cell carcinoma (RCC) and normal renal cultures identified cathepsin D that was subsequently validated in urine samples from 239 patients and healthy and benign disease subjects.

**Results:**

Urinary cathepsin D was found to be significantly associated with overall (OS) (hazard ratio, HR, 1.33, 95%CI [1.09–1.63], *P*=0.005) and cancer-specific survival (HR 1.36, 95%CI [1.07–1.74], *P*=0.013) in RCC patients on univariate analysis. An optimal cut point (211 ng ml^−1^ *μ*molCr^−1^) around which to stratify patients by OS was determined. Five-year OS equal to/above and below this value was 47.0% (95%CI 35.4%, 62.4%) and 60.9% (48.8%, 76.0%), respectively. On multivariable analysis using pre-operative variables, cathepsin D showed some evidence of independent prognostic value for OS (likelihood ratio test *P*-value=0.056) although requiring further validation in larger patient numbers with sufficient statistical power to determine independent significance.

**Conclusion:**

These data establish an important proof of principle and show the potential of proteomics-based studies. Cathepsin D may be of value as a pre-operative urinary biomarker for RCC, alone or in combination.

There are approximately 65 000 new cases of conventional renal cell carcinoma (RCC) in Europe each year, resulting in a substantial economic burden (Ferlay *et al*, 2007; Gupta *et al*, 2008). Despite recent advances in our understanding of the underlying biology, there are still no validated circulating biomarkers of RCC, which may be of value in diagnosis, prognosis or monitoring of patients.

Diagnostic markers, obviating the requirement for biopsy, are desirable, given the increased detection of smaller renal masses, of which approximately 20–25% are found to be benign (Schachter *et al*, 2007). Accurately determining prognosis is important in defining intensity of treatment and follow-up for individual patients. Such efforts have traditionally focused on pathological criteria, based on the surgical specimen (Leibovich *et al*, 2003; Sorbellini *et al*, 2005). However, estimates of risk are relatively wide for individual patients (Leibovich *et al*, 2003; Sorbellini *et al*, 2005) and some elements such as nuclear grade are subject to intra- and inter-observer variability (Al Aynati *et al*, 2003). The possibility of determining outcome pre-operatively has also recently been highlighted, with the definition of six pre-operative variables that predict risk of RCC-specific mortality with a high degree of accuracy (Raj *et al*, 2008; Karakiewicz *et al*, 2009). Such nomograms may influence patient management in terms of treatment modality, surgical strategy and neoadjuvant treatment using suninib or sorafenib for example.

Proteomic technologies hold great potential for the identification of cancer biomarkers (Koomen *et al*, 2008). Circulating protein markers, identified in the blood or urine, could provide more objective information and pre-operatively. However, analysis of such fluids for initial biomarker discovery is challenging because of high salt content, very wide dynamic range of protein concentrations and dominance by a small number of proteins present in high concentrations (Hanash *et al*, 2008). Growth media conditioned by tumour cells in culture can be used to enrich for secreted and shed membrane proteins, which may represent putative circulating markers of disease (Xue *et al*, 2008). Potential candidates can then be analysed in urine or blood and clinical utility assessed. The aim of this study was to carry out a pilot investigation as to whether this approach would successfully identify potential markers for renal cancer, which could then be validated in patient samples for diagnostic or prognostic use and which could form the basis for more intensive larger-scale studies subsequently.

## Materials and methods

### Patient samples

Mid-stream urine samples from 149 untreated patients with clear cell RCC (before nephrectomy), 30 patients with benign urological conditions and 60 healthy controls of similar age and sex were used in the study (Table 1). Immediately post voiding, samples were collected, placed on ice and pH adjusted to 7.0. One mini protease inhibitor cocktail tablet (Roche, Burgess Hill, Sussex, UK) was added per 50 ml. The urine was filtered, centrifuged at 2000 **g** at 4°C for 10 min, aliquotted and stored at −80°C. Samples were collected following ethical approval between May 1999 and June 2007 at St James's University Hospital as part of our prospective sample banking for biomarker studies. All RCC patients except one (biopsy only) subsequently underwent a nephrectomy. Controls were matched for similar length of sample storage and age/gender mix as the patients.

### Cell lines and conditioned medium preparation

The human RCC cell lines HTB46, HTB47, HTB49 (ATCC) and TK10 (NCI Frederick Repository, Washington, USA) were grown in RPMI 1640 medium (HTB49-MEM alpha)/10% v/v FCS/2 mM L-glutamine and maintained at 37°C in an humidified incubator with 5% CO_2_. Ten primary renal cultures (four normal and three matched normal/tumour pairs) from patients with clear cell RCC (grades 2–4; stage III/IV) were generated from nephrectomy samples (normal pole of nephrectomy specimen and areas of tumour, respectively) and characterised by immunocytochemistry to confirm epithelial origin as described earlier (Craven *et al*, 2006b). For this proof of principle study, the strategy involved comparison by 2D PAGE of conditioned media (CM) from HTB49 with HTB49 cell lysate to characterise proteins enriched in CM, which may represent secreted or shed membrane proteins. HTB49 CM was then compared with a CM pool prepared from four normal primary cultures to further highlight potential tumour/normal differences with prioritisation given to those enriched in CM compared with lysate. It was not possible to use the ideal choice of primary matched pair cell lines for the discovery experiments because of limited culture potential and the volumes of CM needed and this also dictated the use of pooled CM as the comparator.

For collection of CM, cells were grown to 70–80% confluency, washed 3 × with HBSS and media replaced with serum-free culture medium supplemented with insulin–transferrin–sodium selenite (Sigma-Aldrich, Poole, Dorset, UK). After 24 h incubation, which had been shown earlier not to affect cell viability, CM was collected, placed on ice and filtered through a 0.2 *μ*m filter to remove cell debris. After addition of protease inhibitor (Complete, Roche; one tablet per 50 ml), supernatants (200 ml) were concentrated using a Gyrosep 300 stirred cell (Intersep, Wokingham, Berks, UK) pressurised at 4 bar with a 10 kDa MWCO ultrafiltration membrane (Millipore, Watford, Herts, UK) with further concentration to 1 ml using a 10 kDa MWCO Amicon Ultra centrifugal filter device (Millipore). The CM was then buffer exchanged into 2D PAGE lysis buffer [12] and stored at −80°C.

### 2D PAGE analysis

Samples (30 *μ*g of protein; triplicate) were subjected to isoelectric focusing on 18 cm pH 3–10 NL IPG strips before separation by SDS–PAGE (10% polyacrylamide), silver staining and analysis, all as described earlier (Craven *et al*, 2006b). Spots upregulated >2-fold within each cell line-normal culture comparison (*P*<0.05, Student's *t*-test) were selected for further analysis. For preparative grade gels, 1 mg protein was used and gels were fixed and stained with PlusOne silver stain using a modified protocol compatible with MS (Yan *et al*, 2000).

### Mass spectrometric sequencing

Excised protein spots were digested with trypsin (Craven *et al*, 2006b). Positive-ion MALDI mass spectra were obtained using an Applied Biosystems 4700 Proteomics Analyser (Applied Biosystems, Manchester, UK) in reflectron mode. Final mass spectra were internally calibrated using tryptic autoproteolysis products or using an internal spike (angiotensin, 1296.6853). Three to ten of the strongest peaks (*S* : *N* >50) in each MS spectrum were selected for CID-MS/MS. The default calibration was used for MS/MS spectra, which were baseline subtracted and smoothed with peak detection using a minimum *S* : *N* of 3 or 5, local noise window of 50 *m*/*z* and minimum peak width of 2.9 bins or as described earlier (Craven *et al*, 2006a). Batch-acquired MS and MS/MS spectral data were submitted to a combined peptide mass fingerprint and MS/MS ion search through the Applied Biosystems GPS Explorer software interface (version 3.5) to Mascot (Matrix Science Ltd, version 2.2.03, London, UK) with criteria as described earlier (Vasudev *et al*, 2008).

### Validation of results by western blotting

Samples of CM (5 *μ*g) were analysed by western blotting using 10% acrylamide gels (Craven *et al*, 2006a). A monoclonal antibody to cathepsin D (clone 49, BD Biosciences (Oxford, UK); 12.5 ng ml^−1^) was used and detection performed using mouse EnVision HRP-conjugated antibody (Dako, Ely, Cambridgeshire, UK; 1 : 200). In the absence of a reliable loading control for CM, parallel Coomassie brilliant blue stained gels were used to check for equal loading.

### Immunoassay for cathepsin D

Urine was thawed, vortexed and microfuged briefly. An in-house sandwich ELISA for cathepsin D was developed and validated. All wash and dilution steps used solution WB (2.5 mM NaH_2_PO_4_, 7.5 mM Na_2_HPO_4_, 500 mM NaCl/0.1% (v/v) Tween 20, pH7.2) unless otherwise indicated. Wells (96-well Nunc Maxisorp plates, VWR, Lutterworth, Leicestershire, UK) were coated with 100 *μ*l of monoclonal anti-cathepsin D antibody (5 *μ*g ml^−1^; Clone 49) in sodium phosphate buffer (2.5 mM NaH_2_PO_4_, 7.5 mM Na_2_HPO_4_, 0.145 M NaCl, pH 7.2) overnight at 4°C and washed 3 × . For the standard curve, human cathepsin D (Sigma-Aldrich) was prepared in doubling dilutions from 8000 ng ml^−1^ to 500 ng ml^−1^. Diluted samples (20 × ) and standards (100 *μ*l) were added to duplicate wells and incubated at RT for 2 h. After washing 5 × , 100 *μ*l of rabbit anti-cathepsin D antibody (10.4 *μ*g ml^−1^; Abcam, Cambridge, UK) in WB/10% (w/v) milk was added to each well and incubated at RT for 2 h. After washing, 100 *μ*l of biotinylated goat anti-rabbit antibody (0.19 *μ*g ml^−1^; Dako) in WB/1% (v/v) mouse serum was added, plates incubated for 1 h, washed and 100 *μ*l of HRP-conjugated streptavidin (0.3 *μ*g ml^−1^; Dako) added to each well. After 30 min and a wash step, 100 *μ*l of TMB substrate (Sigma-Aldrich) was added and colour development stopped after 20 min by addition of 100 *μ*l of 0.5 M sulphuric acid per well and absorbance read at 450 nm.

Samples were analysed blind, randomised throughout and two QC samples were included on all plates. Mean intra-assay CVs for duplicate samples was 12.1% (s.d. 12.4) and mean inter-assay CVs for the QC samples over five consecutive days was 12.8%. Linearity was shown on serial dilutions of four samples. Mean recovery on four urines was 109.3% (s.d. 22.8) for 1500 ng ml^−1^ spikes and 112.1% (s.d. 13.4) for 3000 ng ml^−1^ spikes.

### Statistical analysis

Study design and analysis conformed to ReMARK and STARD guidelines (Bossuyt *et al*, 2003; McShane *et al*, 2005). Sample size was determined for the test of diagnostic potential being powered to detect a fair diagnostic ability of the marker (power 1-*β*=0.90, significance level of *α*=0.05), defined as an AUC of 0.7, such that a minimum of 33 patients in each group were required (Obuchowski 2000). Receiver operating characteristic (ROC) curve analysis was performed to test the diagnostic ability of each marker (Obuchowski 2000). Prognostic value was evaluated as a secondary investigation where DFS (defined as date of recurrence or death from any cause), OS and CSS were examined relative to nephrectomy date. Patients who were still alive or who were lost to follow-up were censored, as were patients who died from causes other than RCC in the CSS analysis. To assess the association of cathepsin D with known prognostic factors, non-parametric methods of testing for association (Kruskal–Wallis and Wilcoxon–Mann–Whitney tests), trend (Cuzick's Wicoxon-like test) and correlation (Spearman's rho) were used where appropriate. Similarly, to assess prognostic ability and the characteristics of the study population with respect to known prognostic factors, the Kaplan–Meier estimate of the survival function, the log rank test and Cox proportional hazards regression were used. These methods were undertaken using Stata 9.2 (StataCorp, College Station, TX, USA). To identify the optimum cut point of cathepsin D for prognosis, an ROC(*t*) curve analysis was undertaken (Heagerty *et al*, 2000).

## Results

### Comparative analysis of CM

**Figure 1:**
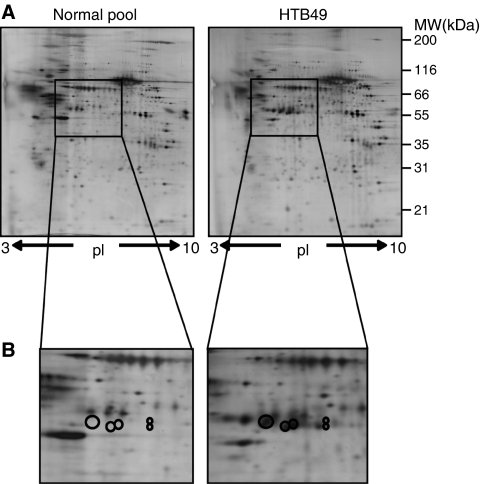
Conditioned media analysed by 2D PAGE. (**A**) A measure of 30 *μ*g of HTB49 and pooled normal renal culture CM were separated on 18 cm pI 3–10 IPG strips in the first dimension. Each sample was run and analysed in triplicate and representative gels are shown. (**B**) The location of cathepsin D as a cluster of spots is shown, which was enriched in CM compared with lysate and increased in RCC CM compared with normal renal primary CM.

One hundred and thirty-three (13%) protein spots were enriched in HTB49 CM in comparison to whole cell lysate, with 27 subsequently identified and accounting for 10 different proteins (Table 2), all of which were predicted to undergo secretion through either classical or non-classical routes (SecretomeP and SignalP) (Bendtsen *et al*, 2004a, 2004b). Gel images and detailed mass spectrometry data of these and a further 40 proteins included for reference purposes are provided as Supplementary data (Supplementary Table 1; Supplementary Figure 1). Comparison of HTB49-derived CM with that from primary normal renal cultures showed 61 (6%) protein spots upregulated in the HTB49 sample. After prioritisation on the basis of enrichment in CM compared with lysate (Table 2), biological relevance, antibody availability and novelty, cathepsin D (Figure 1) was taken forward.

### Western blot validation

**Figure 2:**
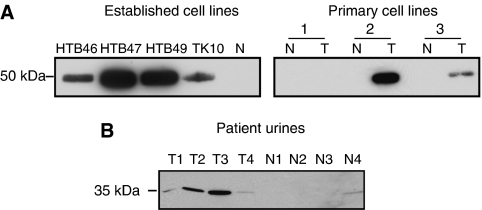
Western blot validation of cathepsin D. (**A**) Samples of CM (5 *μ*g load in each case) were probed for cathepsin D. Expression was seen in all established cell lines examined but absent in one normal sample included for comparison purposes. Increased expression by tumour cultures in two out of three primary tumour (T)/normal (N) matched pairs was also apparent. Parallel Coomassie staining was performed for loading control purposes. (**B**) Urine from four healthy controls and four patients with RCC were probed for cathepsin D. 15 *μ*l of urine was loaded in each case. Uncropped blots are presented in Supplementary Figure 2.

Western blotting confirmed the presence of pro-cathepsin D (50 kDa) in CM from 4/4 established RCC lines, but not the normal CM. In CM from the three matched primary line pairs, cathepsin D was detected in only the tumour samples of two pairs and was absent completely in a third pair (Figure 2A; Supplementary Figure 2). Before immunoassay development, presence of mature cathepsin D (34 kDa) in urine was confirmed in 4/4 samples from RCC patients and only 1/4 healthy control samples (Figure 2B).

### Immunoassay of urinary cathepsin D

All results were normalised by urinary creatinine. Sample storage time varied from 2 to 99 months but comparison of a set of 30 ‘old’ and 30 ‘new’ (average 63.4 months *vs* 7.1 months, respectively) urines from healthy controls found no significant differences. Similar comparisons with patient samples were not feasible because of the confounding background effects of varying disease severity in such a heterogeneous group and will require future longitudinal stability studies.

#### Diagnostic utility

**Figure 3:**
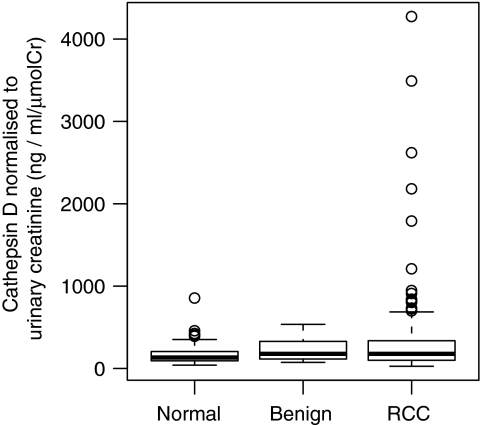
Box-plot of urinary cathepsin D normalised to creatinine. The box extends to the interquartile range (IQR), the line transecting this box representing the median. The bars extend to 1.5 × the IQR and the dots represent outliers that are beyond these limits.

A wider range of cathepsin D concentrations were seen in the RCC group but showed only weak evidence of a significant difference between groups (*P*=0.052 using the Kruskal–Wallis test; Figure 3; Table 3). ROC analysis showed no diagnostic utility with area under the curve being 0.5612, 90% confidence interval (CI) (0.505–0.615) when comparing RCC patients *vs* controls (benign and normal).

#### Association with prognosis

**Figure 4:**
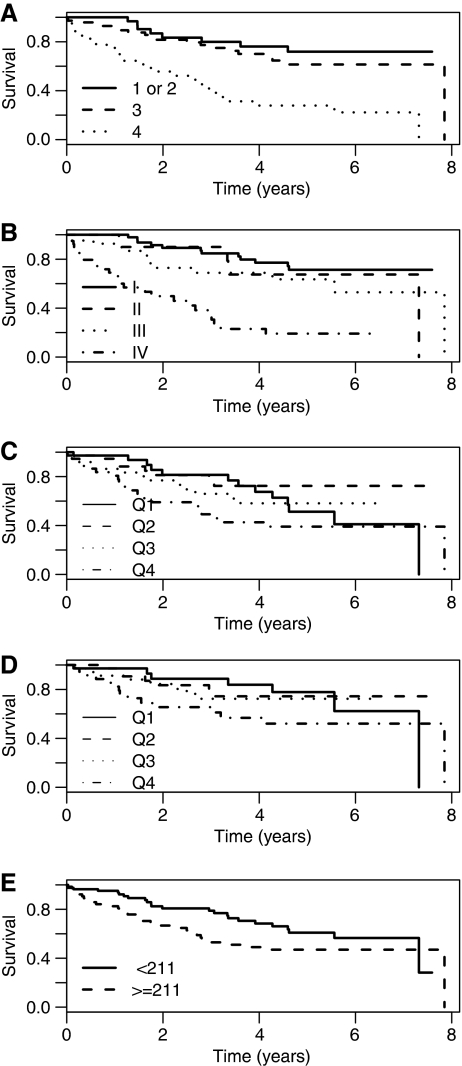
Grade, stage, urinary cathepsin D and survival. Overall survival curves based on (**A**) tumour grade and (**B**) stage validate our patient population. (**C**) Overall and (**D**) cancer-specific survival, respectively, by urinary cathepsin D concentration (normalised for creatinine) considered as quartiles. (**E**) Overall survival based on the optimal cut point of 211 ng ml^−1^ *μ*molCr^−1^ cathepsin D, determined by time-dependent ROC analysis.

Patients with nodal/metastatic disease or sarcomatoid change had significantly higher median urinary cathepsin D levels (Table 3). The median length of follow-up from nephrectomy of those patients still alive was 34 months (range 0–94 months). The representative nature of the population was first confirmed by association of survival with grade and stage (*P*=0.001 for both; Figure 4A and B). On univariate analysis there was evidence of an association between overall survival and cathepsin D (hazard ratio (HR) 1.33, 95%CI [1.09–1.63], *P*=0.005) that is for every rise of 1 unit on the log 2 scale (doubling in concentration of cathepsin D), the hazard of dying increased by 1.3-fold (Figure 4C). There was no evidence of the proportional hazards assumption being violated using the test of Grambsch and Therneau (*P*=0.208). There was also evidence of an association between CSS and cathepsin D (HR 1.36, 95%CI [1.07–1.74], *P*=0.013) (Figure 4D), but not with DFS (HR 1.19, 95%CI [0.93–1.52], *P*=0.168) – although the estimated HR is similar.

Using time-dependent ROC curves, the largest AUC(*t*) was observed for a cut at 18 months (AUC=0.712), when comparing patients who had had an event at 18 months with those who had not. Examining the points of ROC curve gave a best cut-off in terms of sensitivity and specificity for cathepsin D of 211 ng ml^−1^ *μ*molCr^−1^. The 1-year OS estimates (95%CI) for < and ⩾to this cut-off were 95.2% (90.6%, 99.9%) and 80.9% (71.7%, 91.2%), respectively, with corresponding 5-year estimates of 60.9% (48.8%, 76.0%) and 47.0% (35.4%, 62.4%) (Figure 4E). Only 4 patients received adjuvant therapy; 37 patients received IFN and/or IL-2-based treatment in the metastatic setting; 2 patients received sunitinib. However, no difference was observed in the treatment patients received when considered by the cut point for cathepsin D.

To examine further the utility of urinary cathepsin D as a pre-operative marker, the association of cathepsin D with the variables used in the pre-operative nomogram developed by Karakiewicz *et al* (*n*=2474) (Karakiewicz *et al*, 2009), that is CT tumour size, CT T stage and symptoms at presentation was also examined (Table 3). Some evidence of a significant association between increasing cathepsin D concentration and CT T stage (*P*=0.086) was observed when using the non-parametric trend test (confirmed by examining medians in Table 3).

On multivariable analysis, using these pre-operative variables and considering OS, cathepsin D showed some evidence of independent prognostic value (likelihood ratio test (LRT) *P*-value=0.056; Table 4). No evidence was observed for association of cathepsin D with CSS (LRT *P*=0.291) or DFS (LRT *P*=0.103). Metastatic disease, sex, and CT T stage showed independently prognostic value (*P*<0.050), and for other variables HRs were within the 95% CIs reported earlier in a much larger dataset (Karakiewicz *et al*, 2009). The power to show independent prognostic value in this pilot study is low as there are a large number of variables included in the model (nine when considering all levels in categorical variables) and simulation experiments based on this model show that 200 patients would be required at observed event rates to attain a power of approximately 50% and 500 patients to attain a power of approximately 90% with similar HRs to those observed at standard significance levels (Cairns *et al*, in preparation). Hence, a larger study would be required to show strong evidence of an independent prognostic association with cathepsin D.

## Discussion

In this pilot study, we have shown that proteomic analysis of CM from renal cancer cell lines, as described in other diseases (Xue *et al*, 2008), may result in the identification of potential biomarkers such as cathepsin D. Furthermore, although samples from 149 patients with RCC were included, this represents a relatively small validation study with only moderate statistical power for a potential marker with this magnitude of HR, and yet we were able to show the novel finding of an association between urinary cathepsin D in RCC and survival, which has potential clinical application.

Cathepsin D is a lysosomal protease, synthesised as a pre-pro-enzyme. Removal of the signal peptide yields 52 kDa pro-cathepsin D, which undergoes *N*-glycosylation and is then targeted to the lysosome to yield a mature two-chain enzyme consisting of a light (14 kDa) amino-terminal domain and a heavy (34 kDa) carboxyl-terminal domain. The greater amounts of pro-cathepsin D present in RCC CM do not reflect increased transcription (data not shown) but may be translation-related or occur from re-routing of the protein. Such aberrant secretion is present in many cancer types (Liaudet-Coopman *et al*, 2006), in a number of different glycoforms (Capony *et al*, 1989), which may account for the cluster of spots represented by the protein in this study. Pro-cathepsin D is known to function as a mitogen, acting entirely independently of its proteolytic activity (Vetvicka *et al*, 2000; Vashishta *et al*, 2006), although the mechanism mediating these effects is yet to be understood. On the basis of characterisation of the reactivity of the antibodies used in our ELISA, it is likely that the assay measures both precursor and mature forms.

Our current findings are thus biologically plausible. Cathepsin D levels were associated with established features of tumour aggressiveness, such as positive lymph nodes, sarcomatoid change and metastatic disease at presentation as well as CT-defined tumour and overall stage. Increased tissue expression of cathepsin D has been correlated with poorer outcome in a number of other cancers, although inconsistently (Harris *et al*, 2007). In RCC, high expression was associated with a significantly improved OS compared with low expressers (82 *vs* 53 months; *P*=0.03) with independent prognostic significance (Merseburger *et al*, 2005). Tissue cathepsin D did not correlate with tumour characteristics, unlike urinary cathepsin D in our study. These findings seem to contradict our own, but intra-cellular levels of cathepsin D may bear little correlation to the proportion/form of cathepsin D that is externalised. Indeed, unlike tissue, serum cathepsin D levels do not differ between RCC patients and healthy controls (Merseburger *et al*, 2007). Similarly, no diagnostic utility for urinary cathepsin D was found in this study. The source of cathepsin D in healthy control urine is uncertain, but is not thought to originate from serum because differences in glycoforms are apparent (Zuhlsdorf *et al*, 1983) and may reflect release from urothelial cells on turnover.

Urine is accessible but its composition is influenced by many factors such as sex, age, diet, concomitant renal disease and medications, potentially affecting the interpretation of any given test (Barratt and Topham, 2007). However, urinary cancer markers are available and approved by the FDA as exemplified by nuclear matrix protein 22 for bladder cancer. This study illustrates the principle of using CM as an initial source for biomarker discovery, with validation subsequently in biological fluids such as urine. Future work will include increasing the number of cell lines profiled to allow systematic prioritisation of candidate markers based on conserved changes, and increasing the depth of coverage to find potentially more discriminant markers. The potential utility of cathepsin D as a pre-operative marker needs to be explored further using a much larger sample bank to allow a sufficiently powered study for determination of independent prognostic ability and if positive, the inclusion in prognostic models examined. In addition, the relationship between urinary and tissue levels of cathepsin D and the various forms will be investigated with our own work with cell lines indicating a VHL-based regulation of expression (data not shown).

## Figures and Tables

**Table 1:** Details of all patients included in the study

	***N***	**%**
**Characteristic**	**Normal**	
Total	60	100
		
*Sex*		
Male	38	63.3
Female	22	36.7
		
*Age (years)*		
Median (range)	63 (27, 82)	
		
	**Benign**	
Total	30	100
		
*Sex*		
Male	19	63.3
Female	11	36.7
		
*Age (years)*		
Median (range)	58 (34, 84)	
		
*Condition*		
Urinary tract infection	4	13
Benign prostatic hypertrophy	3	10
Renal stone	12	40
Cystic disease	4	13
Benign renal tumour^a^	6	20
Renal atrophy	1	4
		
	**RCC**	
Total	149	100
		
*Sex*		
Male	94	63.1
Female	55	36.9
		
*Age (years)*		
Median (range)	61 (37, 85)	
		
*pTumour diameter (cm)*		
Median (range)	6 (2.0, 15.5)	
		
*CT Tumour diameter (cm)*		
Median (range)	6 (2.0, 21)	
		
*Grade*		
1	3	2.0
2	33	22.2
3	73	49.0
4	40	26.8
		
*Sarcomatoid change*		
No	137	91.9
Yes	12	8.1
		
*pT stage*		
1a	31	20.8
1b	32	21.5
2	14	9.4
3a	27	18.1
3b	42	28.2
4	3	2.0
		
*CT T stage*		
1a	35	27.3
1b	38	29.7
2	31	24.2
3a	19	14.8
4	5	3.9
		
*N stage*		
0	132	88.6
1	17	11.4
		
*M stage*		
0	111	74.5
1	38	25.5
		
*TNM stage*		
I	58	38.9
II	10	6.7
III	42	28.2
IV	39	26.2
		
*Symptoms*		
Local	63	43.2
Asymptomatic	49	33.6
Systemic	34	23.3
		
*Relapse*		
No	76	65.5
Yes	40	34.5
		
*Cancer death*		
No	112	75.2
Yes	37	24.8
		
*Death from any cause*		
No	94	63.1
Yes	55	36.9

Abbreviation: CC=renal cell carcinoma.

a 3 × oncocytoma, 1 × metanephric adenoma, 1 × cystic nephroma, 1 × inflammatory pseudotumour.

**Table 2:** HTB49 CM spot identities

**Name**	**Accession no.**	**No of spots**	**No. of matched peaks**	**No. of unmatched peaks**	**% Coverage**	**MASCOT Score**	**MSMS**	**Status**
IGFBP-rP1	Q16270	1	14	66	45.0	120		SiP
Reticulocalbin 1 precursor	Q15293	1	9	42	42.0	80		SiP^†^
SPARC	P09486	1	9	42	32.0	70		SiP^†^
Vimentin	P08670	1	35	67	71.0	291		SeP
PAI-1	P05121	4	18	30	49.9	157	2	SiP
Transferrin	P02787	8	13	27	30.0	105		
Cathepsin D	P07339	5	9	28	32.1	83		SiP^†^
Nucleobindin 1	Q02818	1	22	56	54.0	172		SiP
BIGH3	Q15582	5	20	33	41.5	166	2	SiP
Complement C3	P01024	1	20	32	23.8	138		SiP

From HTB49 CM, 27 spots (10 proteins) upregulated *vs* whole cell lysate were identified. Where a protein was found in more than one spot, a representative example of scores is shown. Confirmation of peptide mass finger printing (PMF) by MS/MS was undertaken for some spots and this is indicated by the number of significant peptides found. Proteins predicted to contain a signal sequence using SignalP 3.0 are denoted SiP and those predicted to undergo non-classical secretion using SecretomeP are denoted SeP. In the Status column, proteins marked † indicate those proteins upregulated in comparison to normal conditioned media (CM) (further proteins identified are listed in Supplementary Table 1).

**Table 3:** Association of patient and tumour characteristics with urinary cathepsin D by univariate analysis

	**Cathepsin D concentration**		
**Status**	**Median (range) (ng ml^−1^ μmolCr^−1^)**	***P*** **-value (association)**	***P*** **-value (trend)**
Normal	133.2 (40.2, 856.5)	0.052^a^	
Benign	177.6 (73.9, 536.0)		
RCC	178.0 (27.0, 4274.0)		
Control	144.3 (40.2, 856.5)	0.113^b^	
			
*Sex*		0.188	
Male	145.5 (27.0, 4274.0)		
Female	200.0 (40.2, 1209.0)		
			
Age^c^	0.103^c^		0.212
*Grade*		0.854	0.577
1or2	195.0 (27.0, 4274.0)		
3	178.0 (54.0, 3492.0)		
4	172.0 (41.0, 2180.0)		
			
*Sarcomatoid change*		0.032	
No	171.0 (27.0,1789.0)		
Yes	380.0 (73.0,2180.0)		
			
*pT stage*		0.254	0.080
1	172.0 (27.0, 4274.0)		
2	175.0 (70.0, 471.0)		
3	204.5 (48.0, 3492.0)		
4	836.0 (86.0, 1789.0)		
			
*CT T stage*		0.159	0.086
1	161.0 (27.0, 4274.0)		
2	182.0 (27.0, 2180.0)		
3	325.0 (48.0, 912.0)		
4	139.0 (86.0, 802.0)		
			
*N stage*		0.001	
0	165.0 (27.0, 4274.0)		
1/2	362.0 (60.0, 2180.0)		
			
*M stage*		0.034	
0	169.0 (27.0, 4274.0)		
1	251.0 (60.0, 1789.0)		
			
*Overall stage*		0.216	0.043
I	165.0 (27.0,4274.0)		
II	175.0 (70.0,376.0)		
III	164.5 (48.0,3492.0)		
IV	224.0 (60.0,1789.0)		
			
*Symptoms*		0.368	0.190
Local	169.0 (27.0,4274.0)		
Asymptomatic	172.0 (33.0,3492.0)		
Systemic	226.5 (63.0,912.0)		
			
*Tumour size* c			
CT	0.143^c^		0.109
pathology	0.115^c^		0.173

a Refers to test comparing normal, benign and renal cell carcinoma (RCC) group.

b Refers to test comparing control (normal and benign) with RCC group.

c Spearman's rank correlation coefficient.

Age, sex and tumour-related factors relate to RCC group only. *P*-value (association) is result of test comparing distributions in groups under null hypothesis of them being identical and *P*-value (trend) is the result of a test under the null hypothesis of no trend in naturally ordinal groups.

**Table 4:** Univariate and multivariable pre-operative prognostic modelling results for all RCC patients with cathepsin D concentration for OS

	**Univariate analysis**			**Multivariable analysis**		
**Characteristic**	**HR**	**95% CI**	***P-*** **value**	**HR**	**95% CI**	***P-*** **value**
Cath-D (ng ml^−1^ *μ*molCr^−1^)	1.360	(1.069, 1.738)	0.005	1.302	(1.000, 1.696)	0.050
						
Age (yr)	1.007	(0.984, 1.034)	0.607	1.004	(0.969, 1.041)	0.828
						
*Sex*						
M *vs* F	1.655	(0.922, 2.971)	0.091	2.656	(1.057, 6.675)	0.038
						
*CT T stage*						
T2 *vs* T1	1.098	(0.495, 2.436)	0.819	0.525	(0.104, 2.645)	0.435
T3/T4 *vs* T1	6.315	(2.139, 18.642)	0.001	5.450	(1.004, 29.575)	0.049
						
CT max. tumour diameter (cm)	1.155	(1.056, 1.263)	0.002	1.041	(0.821, 1.320)	0.739
						
*M stage*						
M1 *vs* M0	4.623	(2.657, 8.047)	0.001	3.154	(1.178, 8.449)	0.022
						
*Symptoms*						
Local *vs* asymptomatic	2.212	(1.086, 4.505)	0.029	1.230	(0.470, 3.220)	0.674
Systemic *vs* asymptomatic	4.127	(2.008, 8.486)	0.001	1.527	(0.498, 4.683)	0.459

Abbreviations: CI=confidence interval; HR=hazard ratio.
