# Supplementary material for: Pre-operative urinary cathepsin D is associated with survival in patients with renal cell carcinoma
Source: Br J Cancer. 2009 Sep 29;101(7):1175–82. doi: 10.1038/sj.bjc.6605250 (PMC2768081; doi:10.1038/sj.bjc.6605250)
Supplement: Supplementary Figures and Tables [file 6605250x1.doc]

Supplementary Table 1. HTB49 CM spot identities. From HTB49 CM, a total of 67 spots were excised and confidently identified, including 27 spots highlighted as up-regulated in CM vs. whole cell lysate. The MASCOT score for each protein by PMF, as well as the number of matched peptides and sequence coverage are shown. Where confirmation by MS/MS was undertaken, this is indicated by the number of significant peptides found. Proteins predicted to contain a signal sequence using SignalP 3.0^1^ are denoted SiP, and those predicted to undergo non-classical secretion using SecretomeP^2^ are denoted SeP. In the Status column, proteins marked * were identified as up-regulated in comparison to lysate, and those marked with † indicate those proteins up-regulated in comparison to normal CM.

|  | **Name** | **Accession no.** | **No. of matched peaks** | **No. of unmatched peaks** | **% coverage** | **MASCOT**  **Score** | **MSMS** | **mW** | **pI** | **Status** |
| --- | --- | --- | --- | --- | --- | --- | --- | --- | --- | --- |
| 1 | Peptidyl-prolyl isomerase A | P62397 | 10 | 90 | 54.9 | 72 |  | 17881 | 7.7 |  |
| 2 | Peroxiredoxin 1 | Q06830 | 12 | 79 | 56.8 | 92 |  | 22110 | 8.3 | SeP |
| 3 | Triosephosphate isomerase 1 | P60174 | 8 | 34 | 50.8 | 84 |  | 26538 | 6.5 |  |
| 4 | DJ-1 | Q99497 | 7 | 33 | 64.6 | 78 |  | 19891 | 6.3 |  |
| 5 | Glutathione transferase | P09211 | 12 | 81 | 66.0 | 97 |  | 23225 | 5.4 | SeP |
| 6 | Glutathione transferase | P09211 | 10 | 85 | 66.0 | 82 |  | 23225 | 5.4 | SeP |
| 7 | YWHAZ | P63104 | 14 | 47 | 47.2 | 117 |  | 27745 | 4.9 |  |
| 8 | IGFBP-rP1 | Q16270 | 14 | 66 | 45.0 | 120 |  | 29130 | 8.2 | SiP* |
| 9 | Lactate dehydrogenase A chain | P00338 | 11 | 57 | 31.9 | 69 |  | 36689 | 8.3 | SeP |
| 10 | Glyceraldehyde-3-phosphate dehydrogenase | P04406 | 8 | 46 | 43.3 | 70 | 2 | 36053 | 8.6 |  |
| 11 | Glyceraldehyde-3-phosphate dehydrogenase | P04406 | 9 | 37 | 38.5 | 80 | 1 | 36053 | 8.6 |  |
| 12 | Aldose reductase | P15121 | 21 | 71 | 50.5 | 153 |  | 35722 | 6.5 |  |
| 13 | Aldose reductase | P15121 | 17 | 42 | 48.3 | 163 |  | 35722 | 6.5 |  |
| 14 | Lactate Dehydrogenase B chain | P07195 | 13 | 46 | 47.4 | 118 |  | 36638 | 5.7 | SeP |
| 15 | Fructose- bisphosphate aldolase A | P04075 | 21 | 33 | 67.6 | 254 | 2 | 39420 | 8.3 |  |
| 16 | Fructose-bisphosphate aldolase A | P04075 | 13 | 39 | 50.3 | 131 |  | 39420 | 8.3 |  |
| 17 | Fructose- bisphosphate aldolase A | P04075 | 10 | 41 | 37.9 | 86 |  | 39420 | 8.3 |  |
| 18 | Phosphoglycerate kinase I | P00558 | 17 | 36 | 45.7 | 160 | 3 | 44615 | 8.3 |  |
| 19 | Reticulocalbin 1 precursor  SPARC | Q15293  P09486 | 9  9 | 42  42 | 42.0  32.0 | 80  70 |  | 38890  34632 | 4.9  4.7 | SiP*^†^  SiP*^†^ |
| 20 | Vimentin | P08670 | 35 | 67 | 71.0 | 291 |  | 53520 | 5.0 | SeP* |
| 21 | Beta actin | Q96HG5 | 9 | 43 | 35.2 | 76 |  | 41793 | 5.3 |  |
| 22 | Beta actin | Q96HG5 | 18 | 46 | 70.0 | 165 |  | 41793 | 5.3 |  |
| 23 | PAI-1 | P05121 | 18 | 30 | 49.9 | 157 | 2 | 45060 | 6.7 | SiP* |
| 24 | PAI-1 | P05121 | 23 | 81 | 60.4 | 146 |  | 45060 | 6.7 | SiP* |
| 25 | PAI-1 | P05121 | 16 | 41 | 52.8 | 127 |  | 45060 | 6.7 | SiP*^†^ |
| 26 | Alpha enolase | P06733 | 20 | 57 | 63.3 | 169 | 3 | 47038 | 7.0 | SeP |
| 27 | PAI-1 | P05121 | 21 | 51 | 59.3 | 154 | 1 | 45060 | 6.7 | SiP* |
| 28 | Alpha enolase | P06733 | 16 | 53 | 49.9 | 127 | 2 | 47038 | 7.0 | SeP |
| 29 | Transferrin | P02787 | 13 | 27 | 30.0 | 105 |  | 77050 | 6.8 | * |
| 30 | GDP dissociation inhibitor 2 | P50395 | 21 | 33 | 61.7 | 212 |  | 50663 | 6.1 |  |
| 31 | Cathepsin D | P07339 | 9 | 28 | 32.1 | 83 |  | 44552 | 6.1 | SiP*^†^ |
| 32 | Cathepsin D | P07339 | 9 | 36 | 32.1 | 74 |  | 44552 | 6.1 | SiP*^†^ |
| 33 | Transferrin | P02787 | 13 | 50 | 38.6 | 107 |  | 77050 | 6.8 |  |
| 34 | Cathepsin D | P07339 | 14 | 55 | 43.0 | 94 | 1 | 44552 | 6.1 | SiP*^†^ |
| 35 | Cathepsin D | P07339 | 11 | 22 | 50.6 | 111 |  | 44552 | 6.1 | SiP*^†^ |
| 36 | Transferrin | P02787 | 11 | 54 | 34.3 | 80 |  | 77050 | 6.8 | * |
| 37 | Cathepsin D | P07339 | 13 | 51 | 42.4 | 94 | 1 | 44552 | 6.1 | SiP*^†^ |
| 38 | 1.ATP synthase subunit beta  2. Protein disulfide isomerase related protein 5 | Q0QEN7  Q15084 | 21  10 | 48  59 | 63.0  35.0 | 188  72 |  | 48113  48121 | 4.9  4.9 | SiP |
| 39 | Protein disulfide isomerase A3 (ERp60) | P30101 | 19 | 47.4 | 58.0 | 149 | 2 | 56782 | 6.0 | SiP |
| 40 | Transferrin | P02787 | 14 | 57 | 41.0 | 101 |  | 77050 | 6.8 |  |
| 41 | Transferrin | P02787 | 21 | 48 | 45.9 | 178 |  | 77050 | 6.8 | * |
| 42 | Transferrin | P02787 | 9 | 5 | 19.6 | 109 |  | 77050 | 6.8 | * |
| 43 | Aldehyde dehydrogenase 1A1 | P00352 | 14 | 55 | 38.1 | 100 | 3 | 54862 | 6.3 |  |
| 44 | Transferrin | P02787 | 14 | 24 | 30.6 | 120 |  | 77050 | 6.8 | * |
| 45 | Transferrin | P02787 | 17 | 26 | 49.8 | 188 |  | 77050 | 6.8 |  |
| 46 | HSP60 | P10809 | 27 | 67 | 63.1 | 203 |  | 61055 | 5.7 |  |
| 47 | Nucleobindin 1 | Q02818 | 22 | 56 | 54.0 | 172 |  | 53879 | 5.1 | SiP* |
| 48 | Glucose 6 phosphate isomerase | P06744 | 12 | 36 | 27.6 | 92 |  | 63016 | 8.4 |  |
| 49 | Pyruvate Kinase (M1/M2) | P14618 | 19 | 31 | 37.7 | 153 |  | 57937 | 7.9 |  |
| 50 | Pyruvate Kinase (M1/M2) | P14618 | 23 | 44 | 45.3 | 188 | 1 | 57806 | 7.9 |  |
| 51 | BIGH3 | Q15582 | 20 | 33 | 41.5 | 166 | 2 | 74681 | 7.6 | SiP* |
| 52 | Transferrin | P02787 | 20 | 27 | 42.0 | 178 |  | 77050 | 6.8 | * |
| 53 | Transferrin | P02787 | 27 | 39 | 49.0 | 239 |  | 77050 | 6.8 | * |
| 54 | Transferrin | P02787 | 28 | 36 | 48.0 | 243 |  | 77050 | 6.8 | * |
| 55 | HSP 70 protein 8 | P11142 | 23 | 47 | 47.0 | 173 |  | 70898 | 5.4 |  |
| 56 | BiP / GRP78 | P11021 | 29 | 29 | 43.0 | 253 |  | 72333 | 5.1 | SiP |
| 57 | BIGH3 | Q15582 | 23 | 53 | 52.8 | 186 | 2 | 74681 | 7.6 | SiP |
| 58 | Complement C3 | P01024 | 20 | 32 | 23.8 | 138 |  | 187148 | 6.0 | SiP* |
| 59 | BIGH3 | Q15582 | 16 | 37 | 35.5 | 131 | 1 | 74681 | 7.6 | SiP* |
| 60 | BIGH3 | Q15582 | 23 | 49 | 48.5 | 178 | 3 | 74681 | 7.6 | SiP* |
| 61 | BIGH3 | Q15582 | 27 | 47 | 55.3 | 206 | 3 | 74681 | 7.6 | SiP* |
| 62 | BIGH3 | Q15582 | 17 | 28 | 37.4 | 154 |  | 74682 | 7.6 | SiP* |
| 63 | Endoplasmin/Tumour rejection antigen | P14625 | 21 | 29 | 41.9 | 179 | 1 | 92469 | 4.7 | SiP |
| 64 | Alpha actinin 4 | Q96BG6 | 43 | 45 | 53.0 | 362 | 1 | 73619 | 5.2 |  |
| 65 | Complement C3 | P01024 | 13 | 18 | 16.0 | 94 |  | 187164 | 6.0 |  |
| 66 | Alpha glucosidase II alpha subunit | Q14697 | 19 | 15 | 26.7 | 181 |  | 106874 | 5.7 | SiP |
| 67 | Vinculin | P18206 | 23 | 18 | 28.9 | 199 |  | 123799 | 5.5 |  |

1. Bendtsen, J.D., Jensen, L.J., Blom, N., von Heijne, G., and Brunak, S. Feature-based prediction of non-classical and leaderless protein secretion. Protein Eng Des Sel 2004; 17:349-356

2. Bendtsen, J.D., Nielsen, H., von Heijne, G., and Brunak, S. Improved prediction of signal peptides: SignalP 3.0. J.Mol.Biol 2004;340:783-795


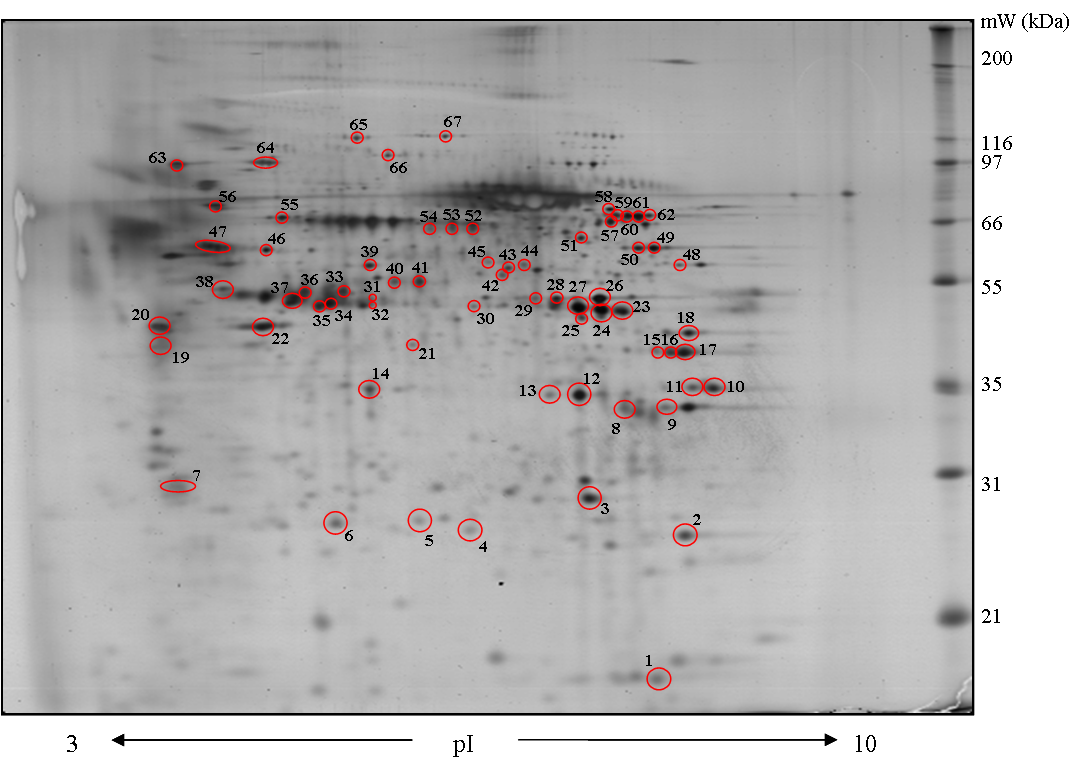


Supplementary Figure 1. Master map of HTB49 CM. A preparative grade gel loaded with 1 mg of HTB49 CM was run and 150 spots excised. The location of the 67 spots identified by tandem mass spectrometry are shown. Corresponding spot identities are shown in Table 1.


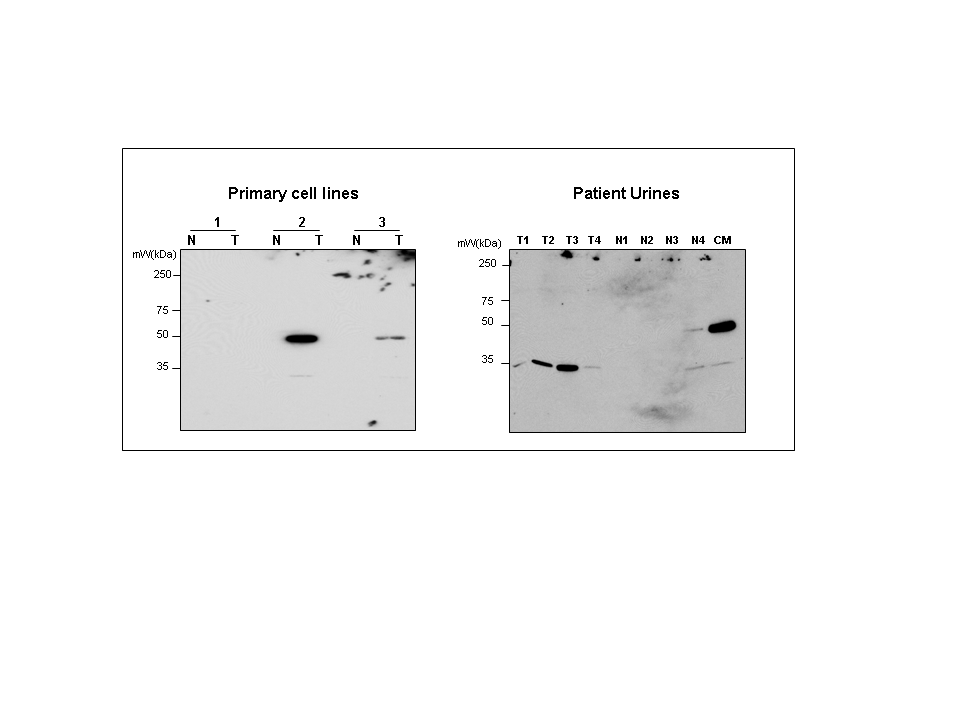


**Supplementary Figure 2. Uncropped Western blots for cathepsin D in primary cell line conditioned media (CM) and patient urines**
